# Supplementary material for: Thermal phenomena and size effects of Mg powder in combustion process
Source: PLoS One. 2024 Sep 16;19(9):e0310185. doi: 10.1371/journal.pone.0310185 (PMC11404785; doi:10.1371/journal.pone.0310185)
Supplement: S1 File — Material Safety Data Sheet of Magnesium Powder. (PDF) [file pone.0310185.s001.pdf]

물질안전보건자료
(Material Safety Data Sheet)

HANA AMT

Regulation UN GHS Rev. 5(2013)에 따라 작성

제품명	Magnesium Powder (Mg Powder)

1. 화학제품과 회사에 관한 정보

가. 제품명	Magnesium Powder(MIL-M-382C, T-III, GR-11/ MIL-M-382C, T-III, GR-12)
나. 제품의 권고 용도와 사용상의 제한	
제품의 권고 용도	산업용 소재 부품, 화학 반응 첨가물
제품의 사용상의 제한	관련법규(위험물 안전관리)에 따름
다. 공급자 정보(수입품의 경우 긴급 연락 가능한 국내 공급자 정보 기재)	
회사명	하나에이엠티 주식회사
주소	본사 : 충북 청주시 청원구 오창읍 각리 1길 75<br>진천공장 : 충북 진천군 문백면 생거진천로 738-3
긴급전화번호	043-211-0047

2. 유해성·위험성

가.유해성·위험성 분류	물반응성 물질 및 혼합물 : 구분1
	자연발화성 고체 : 구분1
	피부 부식성/피부 자극성 : 구분2
	심한 눈 손상성/눈 자극성 : 구분2
	특정표적장기 독성(1회 노출) : 구분3(호흡기계 자극)

나. 예방조치문구를 포함한 경고표지 항목

그림문자

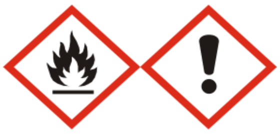

신호어	위험
유해·위험문구	H250 공기에 노출되면 스스로 발화함
	H260 물과 접촉 시 자연발화 가능한 인화성 가스를 발생시킴
	H315 피부에 자극을 일으킴
	H319 눈에 심한 자극을 일으킴
	H335 호흡기계 자극을 일으킬 수 있음
예방조치문구	
예방	P210 열·스파크·화염·고열로부터 멀리하시오 - 금연
	P222 공기에 접촉시키지 마시오.
	P223 물과 접촉하지 않게 하시오.
	P231+P232 불활성 기체 하에서 취급하고, 습기를 방지하시오.
	P261 분진·흄·가스·미스트·증기·스프레이의 흡입을 피하시오.
	P264 취급 후에는 취급 부위를 철저히 씻으시오.
	P271 옥외 또는 환기가 잘 되는 곳에서만 취급하시오.
	P280 보호장갑·보호의·보안경·안면보호)를 착용하시오.
대응	P302+P352 피부에 묻으면 다량의 물로 씻으시오.
	P304+P340 흡입하면 신선한 공기가 있는 곳으로 옮기고 호흡하기 쉬운 자세로 안정을 취하시오.
	P305+P351+P338 눈에 묻으면 몇 분간 물로 조심해서 씻으시오. 가능하면 콘택트렌즈를 제거하시오. 계속 씻으시오.

대응	P312 불편함을 느끼면 의료기관(의사)의 진찰을 받으시오.
	P321 눈에 묻으면 몇 분간 물로 조심해서 씻어내는 등 응급처치를 하시오.
	P332+P313 피부 자극이 생기면 의학적인 조치·조언을 구하시오.
	P335+P334 피부에 묻은 물질을 털어내고, 차가운 물에 담그거나 젖은 붕대로 감싸시오.
	P337+P313 눈에 자극이 지속되면 의학적인 조치·조언을 구하시오.
	P362+P364 오염된 의복은 벗고 다시 사용 전 세척하시오.
	P370+P378 화재 시 불을 끄기 위해 건조모래, 특수파우더를 사용하시오.
저장	P402+P404 건조한 장소에 보관하시오. 밀폐된 용기에 보관하시오.
	P403+P233 용기는 환기가 잘 되는 곳에 단단히 밀폐하여 저장하시오.
	P405 잠금장치가 있는 저장장소에 저장하시오.
	P422 적절한 불활성 기체를 충진하여 보관하시오.
폐기	P501 폐기물관리법에 명시된 내용에 따라 내용물 용기를 폐기하시오.

다. 유해·위험성 분류기준에 포함되지 않는 기타 유해·위험성(NFPA)

보건	0
화재	1
반응성	2

3. 구성성분의 명칭 및 함유량

물질명	마그네슘, 분말 (Magnesium, Powder)
이명(관용명)	MAGNESIUM METAL
CAS 번호	7439-95-4
함유량(%)	98.5 ~ 100%

4. 응급조치요령

가. 눈에 들어갔을 때	눈에 묻으면 몇 분간 물로 조심해서 씻으시오. 가능하면 콘택트렌즈를 제거하시오. 계속 씻으시오.<br>눈에 자극이 지속되면 의학적인 조치·조언을 구하시오.
나. 피부에 접촉했을 때	오염된 옷과 신발을 제거하고 오염지역을 격리하시오<br>경미한 피부 접촉 시 오염부위 확산을 방지하시오<br>피부(또는 머리카락)에 묻으면 오염된 모든 의복을 벗거나 제거하시오.<br>불편함을 느끼면 의료기관(의사)의 진찰을 받으시오.
다. 흡입했을 때	신선한 공기가 있는곳으로 옮기시오.<br>호흡이 힘들 경우 산소를 공급하시오.<br>불편함을 느끼면 의학적인 조치.조언을 구하시오.
라. 먹었을 때	긴급 의료조치를 받으시오<br>물질을 먹거나 흡입하였을 경우 구강대구강법으로 인공호흡을 하지말고 적절한<br>호흡의료장비를 이용하시오.<br>삼켜서 불편함을 느끼면 의료기관(의사)의 진찰을 받으시오.<br>삼켰다면 입을 씻어내시오. 토하게 하려 하지 마시오.
마. 기타 의사의 주의사항	의료인력이 해당물질에 대해 인지하고 보호조치를 취하도록 하시오

5. 폭발·화재시 대처방법

가. 적절한(부적절한) 소화제	건조모래, 특수파우더를 사용하여 소화할 것.<br>물 사용 금지

나. 화학물질로부터 생기는 특정 유해성

화재시 자극성/독성 흄(또는 가스)을 방출함.
물과 접촉 시 자연발화 가능한 인화성 가스를 발생시킴
파우더 또는 알갱이 형태의 경우 공기와 혼합하여 분진 폭발을 일으킬 위험이 있음.
습기, 산과 반응하여 인화성/폭발성 가스를 발생시킴.

다. 화재진압시 착용할 보호구 및 예방조치

구조자는 적절한 보호구를 착용하시오.
지역을 벗어나 안전거리를 유지하여 소화하시오
위험하지 않다면 화재지역에서 용기를 옮기시오.
탱크 화재시 최대거리에서 소화하거나 무인 소화장비를 이용하시오
용기 내부에 물이 들어가지 않도록 하시오

6. 누출사고시 대처방법

가. 인체를 보호하기 위해 필요한 조치사항 및 보호구

분진·흄·가스·미스트·증기·스프레이의 흡입을 피하시오.
매우 미세한 입자는 화재나 폭발을 일으킬 수 있으므로 모든 점화원을 제거하시오.
엎질러진 것을 즉시 닦아내고, 보호구 항의 예방조치를 따르시오.
노출물을 만지거나 걸어다니지 마시오
모든 점화원을 제거하시오
위험하지 않다면 누출을 멈추시오
전문가의 감독없이 청소 및 처리를 하지 마시오
피해야할 물질 및 조건에 유의하시오

나. 환경을 보호하기 위해 필요한 조치사항

환경으로 배출하지 마시오.
수로에 유입되지 않도록 하시오.

다. 정화 또는 제거 방법

청결한 삽으로 누출물을 깨끗하고 건조한 용기에 담고 단단히 밀폐한 뒤 용기를
누출 지역으로부터 옮기시오.
분말 누출시 플라스틱 시트로 덮어 확산을 막고 건조한 상태로 유지하시오.

7. 취급 및 저장방법

가. 안전취급요령

공학적 관리 및 개인보호구를 참조하여 작업하시오
격렬한 반응 및 화재의 가능성이 있으므로 물과 접촉하지 않게 하시오.
불활성 기체 하에서 취급하고, 습기를 방지하시오.
분진·흄·가스·미스트·증기·스프레이의 흡입을 피하시오.
취급 후에는 취급 부위를 철저히 씻으시오.
옥외 또는 환기가 잘 되는 곳에서만 취급하시오.
장기간 또는 지속적인 피부접촉을 막으시오.
피해야 할 물질 및 조건에 유의하시오.

나. 안전한 저장방법

열·스파크·화염·고열로부터 멀리하시오 - 금연
건조한 장소에 보관하시오.
용기는 환기가 잘되는 곳에 단단히 밀폐하여 보관하시오.
피해야 할 물질 및 조건에 유의하시오.

8. 노출방지 및 개인보호구

가. 화학물질의 노출기준, 생물학적 노출기준 등

국내규정	자료없음
ACGIH 규정	자료없음

생물학적 노출기준	자료없음
기타 노출기준	자료없음
나. 적절한 공학적 관리	이 물질을 저장하거나 사용하는 설비는 세안설비와 안전 샤워를 설치하시오.
다. 개인보호구	
호흡기 보호	노출되는 입자상 물질의 물리화학적 특성에 맞는 산업안전보건공단의 인증을 필한 호흡용 보호구를 착용하시오
눈 보호	노출되는 입자상 물질의 물리화학적 특성에 맞는 산업안전보건공단의 인증을 필한 호흡용 보호구를 착용하시오
손 보호	노출되는 입자상 물질의 물리화학적 특성에 맞는 산업안전보건공단의 인증을 필한 호흡용 보호구를 착용하시오
신체 보호	노출되는 입자상 물질의 물리화학적 특성에 맞는 산업안전보건공단의 인증을 필한 호흡용 보호구를 착용하시오

9. 물리화학적 특성

가. 외관	
성상	고체, 분말
색상	은색, 회색
나. 냄새	무취
다. 냄새역치	해당없음
라. pH	해당없음
마. 녹는점/어는점	651 ℃
바. 초기 끓는점과 끓는점 범위	1100 ℃
사. 인화점	자료없음
아. 증발속도	자료없음
자. 인화성(고체, 기체)	자료없음
차. 인화 또는 폭발 범위의 상한/하한	자료없음
카. 증기압	1 Pa (at 428°C)
타. 용해도	물과 반응함
파. 증기밀도	자료없음
하. 비중	1.74
거. n-옥탄올/물분배계수 (Kow)	자료없음
너. 자연발화온도	473 ℃
더. 분해온도	자료없음
러. 점도	1.25 Cp (at 651°C)
머. 분자량	24.3

10. 안전성 및 반응성

가. 화학적 안정성 및 유해 반응의 가능성	공기 및 습기와 접촉시 자연발화하여 자극성/독성 흄을 방출함.<br>산화제 및 기타 여러 물질들과 반응함.<br>물, 산류와 반응하여 인화성/폭발성 가스를 생성함. 화재 및 폭발을 일으킬 위험이 있음.
나. 피해야 할 조건	직사광선, 열, 고온, 습기, 공기와의 접촉
다. 피해야 할 물질	산화제, 물, 산화에틸렌, 금속 산화염, 탄산칼륨.
라. 분해시 생성되는 유해물질	자극성/독성 흄(또는 가스)

11. 독성에 관한 정보

가. 가능성이 높은 노출 경로에 관한 정보	이 물질은 물질의 분진 흡입을 통해 체내로 흡수될 수 있음. (ICSC)
나. 건강 유해성 정보	
급성독성	
경구	LD50 > 2000mg/kg Rat (ECHA)
경피	자료없음
흡입	자료없음
피부부식성 또는 자극성	사람의 피부에 자극을 일으킴. (NLM:HSDB)
심한 눈손상 또는 자극성	사람의 눈에 자극을 일으킴. (NLM:HSDB)
호흡기과민성	자료없음
피부과민성	기니피그를 대상으로 한 Maximisation test 결과 비과민성임. (ECHA)
발암성	
산업안전보건법	자료없음
고용노동부고시	자료없음
IARC	자료없음
OSHA	자료없음
ACGIH	자료없음
NTP	자료없음
EU CLP	자료없음
생식세포변이원성	염색체 이상 시험(Chinese hamster fibroblast, CHL)결과 음성임. (ECHA)
생식독성	자료없음
특정 표적장기 독성 (1회 노출)	직접 접촉시 사람의 눈, 피부 및 호흡기에 자극을 일으킴. (NLM:HSDB)
특정 표적장기 독성 (반복 노출)	장기간 또는 반복적으로 분진에 노출될 경우 폐에 영향을 미칠 수 있음. (ICSC)
흡인유해성	자료없음
기타 유해성 영향	자료없음

12. 환경에 미치는 영향

가. 생태독성	
어류	자료없음
갑각류	LC50 64.7 ㎎/ℓ 96 hr Gammarus lacustris (ECOTOX)
조류	자료없음
나. 잔류성 및 분해성	
잔류성	자료없음
분해성	자료없음
다. 생물농축성	
농축성	자료없음
생분해성	자료없음
라. 토양이동성	자료없음
마. 기타 유해 영향	자료없음

13. 폐기시 주의사항

가. 폐기방법	폐기물관리법에 명시된 경우 규정에 따라 내용물 및 용기를 폐기하시오.
나. 폐기시 주의사항	폐기물관리법에 명시된 내용에 따라 내용물 용기를 폐기하시오.

14. 운송에 필요한 정보

가. 유엔번호(UN No.)	1418
나. 적정선적명	마그네슘분말 또는 마그네슘 합금분말(MAGNESIUM POWDER or MAGNESIUM ALLOYS POWDER)
다. 운송에서의 위험성 등급	4.3(4.2)
라. 용기등급	I

마. 해양오염물질	자료없음
바. 사용자가 운송 또는 운송수단에 관련해 알 필요가 있거나 필요한 특별한 안전대책	
화재시 비상조치	F-G
유출시 비상조치	S-O

15. 법적규제 현황

가. 산업안전보건법에 의한 규제	
나. 화학물질관리법에 의한 규제	해당없음
다. 위험물안전관리법에 의한 규제	2류 마그네슘 500㎏
라. 폐기물관리법에 의한 규제	해당없음
국내규제	
기타 국내 규제	해당없음
국외규제	
미국관리정보(OSHA 규정)	해당없음
미국관리정보(CERCLA 규정)	해당없음
미국관리정보(EPCRA 302 규정)	해당없음
미국관리정보(EPCRA 304 규정)	해당없음
미국관리정보(EPCRA 313 규정)	해당없음
로테르담협약물질	해당없음
스톡홀름협약물질	해당없음
몬트리올의정서물질	해당없음
EU 분류정보(확정분류결과)	F; R15-17
EU 분류정보(위험문구)	R15, R17
EU 분류정보(안전문구)	S2, S7/8, S43

16. 그 밖의 참고사항

가.자료의 출처	산업안전보건공단(KOSHA)<br>한국소방산업기술원(K F I)<br>국립환경과학원 화학물질정보시스템(NCIS)<br>TOXNET(United States National Library of Medicine)<br>ICSCs(International Chemical Safety Cards)<br>ECHA(European Chemicals Agency)<br>ECOTOX Database
나. 최초작성일	2013년 06월 03일
다. 개정횟수 및 최종 개정일자	
개정횟수	7회
최종 개정일자	2021년 2월 10일
라. 기타	이 MSDS는 작성시 당사의 전문자료 및 최신 정보 등에 기초하였으며 제공하는 화학물질의 유해.위험성 분류결과는 인용된 참고자료에 따라 차이가 발생할 수 있습니다. 또한 이 자료는 품질을 보증하는 것이 아니며 물질의 안전에 대한 전반적인 참고자료로 사용하시기 바랍니다. 당사 MSDS는 해당 제품을 공급받아 사용하는 취급자가 주의사항 등을 숙지한 후 사용할 수 있도록 합니다. 또한 판매 및 대여 등 영리목적으로는 사용할 수 없음을 알려드립니다.

물질안전보건자료(MSDS)와 해당제품에 대한 문의사항은 아래 주소로 연락주시기 바랍니다.

* 주소	본사 :	충북 청주시 청원구 오창읍 각리 1길 75
	진천공장 :	충북 진천군 문백면 생거진천로 738-3
* 전화번호		043-211-0047
* 팩스		043-753-7007
* 홈페이지		www.hanaamt.com

HANA AMT
