# Supplementary material for: Thermal phenomena and size effects of Mg powder in combustion process
Source: PLoS One. 2024 Sep 16;19(9):e0310185. doi: 10.1371/journal.pone.0310185 (PMC11404785; doi:10.1371/journal.pone.0310185)
Supplement: Attachment — Submitted filename: reviewer-2.pdf [file pone.0310185.s003.pdf]

Date: August 4, 2024

PLOS ONE

Dear Reviewer 2:

Thank you for giving us the opportunity to submit a revised draft of the manuscript "Thermal Phenomena and Size Effects of Mg Powder". We appreciate to insightful comments on and valuable improvements on our manuscript. We have incorporated most of the suggestions made by the reviewer. Those changes are highlighted within the manuscript.

Comment 1: However, this description must be reorganized because some of the data regarding the research methodology was discussed in the Introduction section, and detailed data was not provided in the Materials and Methods section.

-> Response 1: Additional information on the experimental substance magnesium and the experimental method were explained.

Page 2, Line 43 ~ 47

> ISO-7167 enacted that all particles should be able to pass a sieve with 387 μm mesh size, and a minimum of 80 \% of the powder must be retained in a sieve with 150 μm mesh size [13]. For this reason, the particle size was selected step by step based on the magnesium particle size of 150 μm used in the ISO 7165 fire extinguisher performance test for metal fires [16].

Page 2, Line 49 ~ 56

> This study investigates the phenomenon of Mg metal fire based on thermal and flow visualization because the smoke cannot be ocularly observed during Mg metal burning. The experimental equipment comprises a diode-pumped solid-state (DPSS) laser, a high speed camera, and an IR camera (FLIRA655sc). After the DPSS laser forms a 2D plane, a CCD camera is used to capture the image flowing on the plane. Additionally, the combustion velocity of Mg was measured using a combustion velocity measurement device based on the particle size. The characteristics of the combustion product particles were analyzed using SEM (model: S-4800 made by Hitachi).

Comment 2: This mainly concerns the characterization of the combustion product particles using SEM. A description of the conditions for testing the structure of Mg particles should be provided.

-> Response #2: We added a detailed description of the experimental equipment including SEM used in the experiment.

Page 2, Line 49 ~ 56

> This study investigates the phenomenon of Mg metal fire based on thermal and flow visualization because the smoke cannot be ocularly observed during Mg metal burning. The experimental equipment comprises a diode-pumped solid-state (DPSS) laser, a high speed camera, and an IR camera (FLIRA655sc). After the DPSS laser forms a 2D plane, a CCD camera is used to capture the image flowing on the plane. Additionally, the combustion velocity of Mg was measured using a combustion velocity measurement device based on the particle size. The characteristics of the combustion product particles were analyzed using SEM (model: S-4800 made by Hitachi).

Comment 3: Results and discussion part concerning the results obtained for the combustion characteristics and fire development of Mg through thermal and flow visualization is sufficiently detailed.

-> Response 3: We have rewritten the contents in the Results and Discussion section. We have written additional content in the Combustion Velocity subsection. We have written a new paragraph that can supplement the content in the conclusion.

Page 4, Line 87 ~ 95 : Results and Discussion

Fig 04 show infrared images of the temperature reached after ignition and thermal images depicting the Mg combustion process over time. Owing to the high-temperature conditions under which the experiment is conducted, the temperature range spans from 373.1K to 967.4K to observe heat flow phenomena during combustion. The images show the flame spreading due to the combustion heat. There was a rapid burning accompanied by a white flame and white smoke. Upon analyzing the temperature distribution through the infrared thermal images, we observed that the peripheral areas where combustion is accompanied by flame show a temperature distribution above 967.4 K.

Page 8, Line 157 ~ 166

After measuring the combustion rate according to the particle size, the time for the combustion length to reach 30 mm was 90 seconds for 150 µm, 60 seconds for 100 µm, and 45 seconds for 75 µm. The average combustion length for each size was 32 mm for 150 µm, 35 mm for 100 µm, and 45.7 mm for 75 µm. This indicates that the smaller the particle size, the faster the combustion and diffusion rate. Additionally, in Fig 4 and Fig5, a rapid increase in temperature occurred at approximately 37 seconds and reached the maximum temperature at approximately 48 seconds. As shown in Fig 7, this means that magnesium changes to a vapor state through a smoldering process after ignition, generating a flame and rapid combustion.

Comment 4: However, the caption for Fig. 9 is missing. Moreover, the description of the SEM results should also include a reference to Fig. 1 illustrating the lack of agglomeration of Mg particles, which is less visible in Fig. 9.

-> Response 4: We comprehensively analyzed Fig 1 and Fig 8 and wrote additional content.

Page 8, Line 137 ~ 147 : Combustion velocity

Magnesium undergoes melting, evaporation, and combustion processes due to heat. During these processes, magnesium goes through a melting process and combines with oxygen in the air to produce MgO, and reacts with nitrogen to produce Mg3N2. In addition, a reaction occurs to generate MgOH by combining with some atmospheric moisture. In this melting and chemical reaction process, the reaction rate and amount of reaction vary depending on the particle size, leading to different types of combustion products and particle shapes [18, 19]. Due to this phenomenon. Unlike pure magnesium, which showed a non-uniform but round shape as shown in Fig 1b and Fig 1c, after combustion, it is believed that various inconsistent changes in shape appeared depending on the size of the particles. There is no significant difference in the transformation of magnesium particles by the high temperature heat of combustion.

Fig 9 is renamed Fig 8.

Fig 1, 3, 8 Replaced with high-resolution image (eps format)

Fig 5. Create a new graph (eps format)

Thank you for your consideration. We look forward to hearing from you.

Name of the corresponding author: Jung Kyu Park

Telephone: +82-10-4899-6699

Mailing address: 1007, Hoguk-ro, Pocheon-si, Gyeonggi-do, 11159, Republic of Korea

E-mail: jkpark@daejin.ac.kr
